# Comparing interval estimates for small sample ordinal CFA models

**DOI:** 10.3389/fpsyg.2015.01599

**Published:** 2015-10-30

**Authors:** Prathiba Natesan

**Affiliations:** Department of Psychology, University of North TexasDenton, TX, USA

**Keywords:** Bayesian, ordinal data analysis, confirmatory factor analysis, confidence intervals, simulation, structural equation modeling, Markov chain Monte Carlo

## Abstract

Robust maximum likelihood (RML) and asymptotically generalized least squares (AGLS) methods have been recommended for fitting ordinal structural equation models. Studies show that some of these methods underestimate standard errors. However, these studies have not investigated the coverage and bias of interval estimates. An estimate with a reasonable standard error could still be severely biased. This can only be known by systematically investigating the interval estimates. The present study compares Bayesian, RML, and AGLS interval estimates of factor correlations in ordinal confirmatory factor analysis models (CFA) for small sample data. Six sample sizes, 3 factor correlations, and 2 factor score distributions (multivariate normal and multivariate mildly skewed) were studied. Two Bayesian prior specifications, informative and relatively less informative were studied. Undercoverage of confidence intervals and underestimation of standard errors was common in non-Bayesian methods. Underestimated standard errors may lead to inflated Type-I error rates. Non-Bayesian intervals were more positive biased than negatively biased, that is, most intervals that did not contain the true value were greater than the true value. Some non-Bayesian methods had non-converging and inadmissible solutions for small samples and non-normal data. Bayesian empirical standard error estimates for informative and relatively less informative priors were closer to the average standard errors of the estimates. The coverage of Bayesian credibility intervals was closer to what was expected with overcoverage in a few cases. Although some Bayesian credibility intervals were wider, they reflected the nature of statistical uncertainty that comes with the data (e.g., small sample). Bayesian point estimates were also more accurate than non-Bayesian estimates. The results illustrate the importance of analyzing coverage and bias of interval estimates, and how ignoring interval estimates can be misleading. Therefore, editors and policymakers should continue to emphasize the inclusion of interval estimates in research.

## Introduction

Ordinal data is frequently used in educational and behavioral research. Several methods such as asymptotically generalized least squares (AGLS) and robust maximum likelihood (RML) have been recommended specifically for ordinal structural equation models (SEMs, e.g., Muthén, 1984; Jöreskog, 1994; Yang-Wallentin et al., 2010; Yuan et al., 2011). These methods generally work well with sufficiently large samples. Data collection, however, is an expensive endeavor. Consequently behavioral researchers often work with small sample data. Even with these techniques, many challenges to model estimation remain in small samples (Yuan et al., 2011; Bandalos, 2014). For instance the polychoric correlation matrices are more likely to be non-positive definite when the sample size is insufficient or the number of variables is large. This leads to frequent non-converging solutions in RML (Babakus et al., 1987; Yuan et al., 2011). Bandalos (2014) and Yang-Wallentin et al. (2010) compared various AGLS approaches for ordinal SEMs in small sample situations. Even these methods fail to produce acceptable estimates for sample sizes smaller than 200. Bayesian methods have gained some popularity in the recent times due to their advantage with small sample data and minimal data distribution assumptions. Lee and Song (2004a,b) and Lee et al. (2010) have investigated the use of Bayesian methods for ordinal structural equation models. Yet, Bayesian methods remain underutilized due to steep learning curve, longer estimation times, lack of inclusion of Bayesian methods in educational research curriculum, and fewer software solutions.

Several researchers (e.g., Gardner and Altman, 1986; Thompson, 2002; Gelman et al., 2004; Cumming and Finch, 2005; Cumming, 2012) and the American Psychological Association (APA, 2010) have emphasized the necessity of reporting interval estimates along with point estimates. This provides a better understanding of the uncertainty and magnitude of parameter estimates. The lack of precision of a sample statistic can be clearly shown using an interval estimate (Gardner and Altman, 1986). Most studies that compare RML and AGLS for SEMs have focused only on point estimates and standard errors of parameters. However, overly small standard errors can result in higher probability of incorrectly rejecting the null hypothesis. Researchers may falsely believe that the estimates have less uncertainty because of shorter confidence intervals. The coverage rates of confidence intervals may also be smaller than expected. Overly large standard errors (or wide confidence intervals) indicate lack of precision in a statistical estimate. For instance, consider a confidence interval for Pearson's correlation to be [−0.99, 0.99]. This is as good as having no estimate at all because it almost covers the mathematical range of Pearson's correlation. Therefore, standard errors need to be examined not just for their magnitude but also for their accuracy and impact on Type-I error. In sum, comparisons of estimation methods must always include analysis of both interval and point estimates.

Interval estimates (confidence and credibility) must be examined in addition to standard errors. Coverage rate is the percentage of intervals of the statistical estimate that contain the true parameter value. A 95% confidence interval will contain the true parameter value 95% of the time when resampled. The probability of Type-I error increases when the coverage rate falls below this value. However, examining coverage rates alone can be misleading (Jennings, 1986, 1987; Schall, 2012). Instead coverage rates should be reported along with how many times the parameter value was over-estimated and under-estimated. For instance, consider a confidence interval that always overestimates the true value when it does not contain the true value. Then this confidence interval systematically overestimates the true value. An unbiased estimator is equally likely to be above or below the true value when the data is normal. Therefore, it is necessary to compare the performance of these various estimation methods, not just, with respect to their point estimates, but with respect to their interval estimates.

The purpose of the present study is to compare the interval estimates of factor correlations of the simplest ordinal confirmatory factor analysis (CFA) model from five different estimation methods in small sample cases. Width, coverage rates, and direction of bias of AGLS, RML, and Bayesian interval estimates and standard errors of estimates for ordinal CFA models were studied for small sample cases. Confidence intervals were examined for AGLS and RML methods and credibility intervals were examined for Bayesian methods. In Bayesian methods, prior specification allows researchers to incorporate systematic information about a parameter into the current estimation (Fox, 2010). Although, the effect of priors on parameter estimates is quite minimal in large samples, priors could have a considerable impact in small samples. Therefore, priors should be investigated when studying small sample cases. A relatively less informative prior gives very less information about a parameter. In the present study a uniform distribution ranging from -1 to 1 was considered a relatively less informative prior for factor correlation. This prior assigns equal probability value for every real number between −1 and 1. An informative prior gives some information about a parameter. This could be based on previous research or substantive reasoning (e.g., happiness is positively correlated with positive emotional quality of life). The informative prior considered in the present study was a uniform distribution ranging from 0 to 1 for the factor correlation where the researcher believes that the relationship between the factors is positive. The priors were varied only for factor correlations. The rest of the priors were fixed to be the same between the conditions in order to isolate the effect of the relatively less informative factor correlation prior on the factor correlation estimates.

Performance under various data conditions such as sample size, prior specification for Bayesian estimation, and distribution shape of factor scores were studied. The results:

- inform the method(s) and data conditions required for accurate point and interval estimation of parameters in ordinal CFA, and
- illustrate the importance of examining width, coverage rate, and direction of bias of confidence intervals in statistical simulation studies.

To my knowledge, no study has systematically compared the coverage rates, widths, and direction of bias of Bayesian, AGLS, and RML interval estimates in ordinal CFAs for small sample cases. Lee et al. (2010) used probit and logit links to model non-linear structural equation models for dichotomous data. They found that informative priors can be used to increase the accuracy of parameter estimates. Although, Lee and Song (2004a) showed that Bayesian estimation outperformed ML in estimating SEMs for small sample cases, their study was conducted for intervally-scaled data and compared only these two estimation methods. Their study did not investigate coverage rates nor the impact of prior specification on parameter recovery.

The present study answers the research question: How do the performance of Bayesian credibility intervals and RML and AGLS-based confidence intervals compare with respect to width, coverage, and direction of bias? A simple two-factor CFA model with five items per factor was considered. Two factor score distributions (multivariate normal, multivariate mildly skewed), six sample sizes (*n* = 2df, 3df, 4df, 5df, 10df, 15df), three factor correlations (low = 0.2, medium = 0.5, high = 0.8), and 2 prior distributions for the correlation (ξ) between the latent variables [ξ~ Unif(-1,1), ξ~ Unif(0,1)] were studied. A brief overview of ordinal CFA and commonly used estimation methods follows.

### Ordinal CFA

Let *y*_1_, *y*_2_, …*y*_*p*_ be *p* ordinal variables. A continuous variable $y_{i}^{*}$ with range (−∞, ∞) is assumed to underlie each corresponding ordinal variable *y*_*i*_. Variables $y_{i}^{*}$ and *y*_*i*_ are mapped to each other based on strictly increasing threshold parameters τ_*k*_ as:

(1) $$y_{i}=\{\begin{array}{c} 1,\tau_{0}\leq y_{i}^{*}<\tau_{1}\\ 2,\tau_{1}\leq y_{i}^{*}<\tau_{2}\\ \vdots \\ c,\tau_{c-\text{1}}\leq y_{i}^{*}<\tau_{c} \end{array}$$

where, τ_0_ = −∞ and τ_*c*_ = ∞ for item *i* with *c* categories. Consider a CFA model of sample size *n*, given as:

(2) $$\begin{array}{r} y_{i}^{*}\;=\;\mu \;+\;\Lambda \omega_{i}\;+\;\epsilon_{i},i=1,\ldots ,n, \end{array}$$

where, $y_{i}^{*}$ is the (*p* × 1) unobservable manifest random vector, μ is the (*p* × 1) vector of intercepts, Λ is the (*p* × *q*) factor loading matrix, ω_*i*_ is a (*q* × 1) latent random vector (factor scores), ϵ_*i*_ is a (*p* × 1) random vector of error measurements independent of ω_*i*_. Let ϕ and θ be the covariance matrices of ω and ϵ, respectively. When the unique factors are uncorrelated, ϕ is a diagonal matrix. If ϕ is a correlation matrix, the covariance matrix of y^*^ is Σ and given as,

(3) $$\begin{array}{r} \Sigma =\Lambda \phi \Lambda^{\prime }\;+\;\Theta . \end{array}$$

Given that the underlying variables $y_{i}^{*}$ have variances equal to 1, if *I* is the identity matrix of size *p* × *p*,

(4) $$\begin{array}{r} \Theta =I\;-\;diag\left(\Lambda \phi \Lambda^{\prime }\right), \end{array}$$

and

(5) $$\begin{array}{l} \Sigma \left(\Lambda ,\Phi \right)=\Lambda \Phi \Lambda^{\text{′}}\;+\;I-diag\left(\Lambda \Phi \Lambda^{\text{′}}\right). \end{array}$$

### Maximum likelihood

Maximum likelihood (ML) estimation has been frequently used to estimate model parameters for ordinal data although it is least justified for use with ordinal data (Yang-Wallentin et al., 2010). ML with polychoric correlations provides adequately accurate estimates of parameters (based on bias and mean squared error), but it also produces the most non-convergences (Babakus et al., 1987). Polychoric correlation matrix (PCM)-based ML parameter estimates may be acceptable at *n* = 300 but non-convergence in small samples is a frequent issue. This is because polychoric correlations are obtained from different marginals in the ML approach, and are more likely to be non-positive definite when the sample size is insufficient or the number of variables is large (Yuan et al., 2011). Additionally, distribution shape of ordinal data, sample size, and fitting function all affect the convergence rate of PCM-based estimates (Rigdon and Ferguson, 1991). Most ML approaches assume the observed variables to be multivariate normally distributed, but often ordinal and extreme response data do not exhibit multivariate normality. This problem is exacerbated in small samples or extreme response data in sensitive questionnaires. Ignoring non-normality of data can produce seriously erroneous parameter and confidence interval estimates (Boomsma, 1982; Chou et al., 1991).

Improper or inadmissible solutions such as negative error variances are another issue in using ML for small sample ordinal data CFA. Anderson and Gerbing (1984) and Boomsma (1982; 1985) demonstrated that the chance of negative variance estimates in models based on ordinal data increase with a decrease in the sample size, the number of indicator variables per factor, and the factor pattern coefficient values. For instance, Anderson and Gerbing (1984) showed that for 2 latent variables with 2 observed variables each, 10–86% of the ML-based solutions were inadmissible for samples with sizes between 50 and 150. Similarly, for models with 2 latent variables, 3 observed variables each, samples 150 or less produced 16–53% inadmissible ML-based solutions.

### Asymptotically generalized least squares

Asymptotically generalized least squares (AGLS)-based approaches produce generalized least squares estimates using asymptotic covariance matrices (ACMs) as weight matrices (e.g., Christofferson, 1975; Muthén, 1978). Based on Browne's (1984) asymptotically distribution free (ADF) estimator, Muthén (1984) developed categorical variable methodologies (CVM) while Jöreskog (1990) used the asymptotic covariance matrix of polychoric correlations to fit models for ordinal data. Jöreskog's approach uses marginal frequencies to estimate thresholds and pairwise frequencies to estimate polychoric correlations when holding the thresholds constant. Both these approaches yield similar results when used with WLS (Yang-Wallentin et al., 2010). Lee et al. (1990) presented an AGLS approach for ordinal SEM by estimating thresholds and polychoric correlations using ML. All least squares methods use a two-step procedure where the PCM and ACM are estimated in the first step. In the second step, matrices Λ and Φ in Equations (3–5) are fitted to the PCM *r* by minimizing the fit function

(6) $$\begin{array}{r} F\left(r,\Lambda ,\Phi \right)={\left[r\;-\;\rho \left(\Lambda ,\Phi \right)\right]}^{\prime }V\left[r\;-\;\rho \left(\Lambda ,\Phi \right)\right]. \end{array}$$

In Equation (6), *V* is a positive weight matrix and ρ(Λ, Φ) is a vector of the elements of Λ*ΦΛ*′ below the diagonal. Based on the choice of the weight matrix, three least squares approaches are commonly used: weighted least squares (WLS), unweighted least squares (ULS), and diagonally weighted least squares (DWLS). The ULS approach uses an identity matrix as the weighting matrix (i.e., no weighting), WLS uses the inverse of the ACM and DWLS uses only the diagonal elements of the inverse of the ACM. ULS, ML, and DWLS methods that use PCM combined with ACM are named robust unweighted least squares (RULS), robust maximum likelihood (RML), and robust diagonally weighted least squares (RDWLS), respectively.

Both Muthén's and Jöreskog's approaches yield almost identical results when applying the weight matrices in the weighted least squares (WLS) approach for ordinal data. Although the sample covariance matrix does not have to be positive definite, the ACM has to be positive definite for WLS. WLS requires the sample sizes to be fairly large but the number of variables to be relatively small. This is because the weight matrix is extremely unstable for smaller samples (Yang-Wallentin et al., 2010) resulting in frequent convergence issues. Hu et al. (1992) suggested using samples greater than 5000 when using WLS. Olsson et al. (2000) suggested *n* > 1000 for WLS estimation with non-normal data. Several studies have also reported that WLS estimators produce incorrect standard errors and chi-squares (e.g., Potthast, 1993; Dolan, 1994; Bentler, 1995; DiStefano, 2002; Flora and Curran, 2004). Non-convergence in WLS worsens for complex models with a large number of observed variables (Muthén and Kaplan, 1992) and non-normality (Bandalos, 2014).

DWLS avoids the instability problem of WLS matrix in small samples by using only the diagonal weight matrix for parameter estimation but the entire weight matrix to estimate standard errors. DWLS estimation has been shown to produce overall acceptable Type-I error rates for samples as small as 200 (Muthén et al., 1997). ULS does not require a positive definite sample covariance matrix, is faster, and provides good point estimates but not good standard errors (Wothke, 1993).

Forero et al. (2009) showed that DWLS and ULS estimation of ordinal CFAs had an average convergence rate of 71% and 57%, respectively for a sample size of 200. Yang-Wallentin et al. (2010) compared the performance of RULS, RML, and RDWLS. Neither of the three showed uniformly better performance. These findings are supported by Bandalos (2014) who compared RML, RDWLS, and WLS for categorical non-normal data under model misspecification. Although RULS produced acceptable range of standard errors and small root mean square errors (RMSE), it ran into convergence issues for small samples (*n* < 200). Even if the original data are normal, the distribution of the sample covariance matrix approaches normal only when the sample sizes are large. This is a reason for non-convergence (Lee and Song, 2004a). Beauducel and Herzberg (2006) reported that RDWLS standard errors are uniformly lower than those from ML. These investigations focused on whether and how small the standard errors are. However, an equally important question is whether these standard errors are adequately controlling Type-I error rates and providing reliable confidence intervals.

### Bayesian estimation

Sampling-based Bayesian methods depend less on asymptotic theory and therefore, can be particularly useful with non-normality in small samples (Scheines et al., 1999; Ansari and Jedidi, 2000; Ansari et al., 2000; Dunson, 2000). Prior information can be included in a meaningful manner to obtain more accurate estimates of parameters for small samples (Lee and Song, 2004a,b). Bayesian parameter estimates allow probabilistic interpretation (e.g., posterior distribution) as opposed to single point estimates in non-Bayesian methods (Gelman and Rubin, 1992; Gelman, 1996). This makes interpretation of Bayesian credibility intervals more straightforward than confidence intervals in frequentist approaches (Gelman et al., 2004; Lynch, 2010). Complex statistical models can be more efficiently estimated using Bayesian methods. Therefore, Bayesian estimation could be potentially advantageous in estimating ordinal CFAs. Complexity in writing Bayesian algorithms and longer estimation time in widely available software programs such as OpenBUGS and JAGS are some practical disadvantages of Bayesian methods. Readers may refer to Lee and Song (2004a,b), Lee (2007), and Shi and Lee (1998) for discussion on Markov chain Monte Carlo (MCMC) estimation of SEMs and CFAs.

The present study considers the MCMC algorithm, and more specifically the Gibbs sampler for Bayesian estimation of ordinal CFAs. Prior distributions must be carefully chosen in small samples for Bayesian estimation because their impact on the posterior distributions increases in small samples. Therefore, two prior distributions—relatively less informative and informative priors were studied. Following Shi and Lee (2000) and Song and Lee (2001, 2002), model identification restrictions were placed to identify the model. The mean and variance of each component of y^*^ in Equation (2) were fixed to zero and one, respectively.

#### Gibbs sampler

The Gibbs sampler, which is the most basic MCMC method, samples the conditional distribution of a parameter given the current value of all other parameters. This process is repeated for each parameter and estimates are updated with each iteration. The Gibbs sampling algorithm (Geman and Geman, 1984; Gelfand and Smith, 1990; Albert, 1992) implemented in the present study follows: Let, Ω = (κ, α) be the vector of parameters where κ is the matrix of the unknown parameters μ, Λ, Φ, and Θ and α is the vector of unknown thresholds corresponding to *y*_(*c*)_, and α = (α_1_, α_2_, …, α_*p*_), and *Y* = (*y*_1_, …, *y*_*n*_) the observed categorical response data matrix. Let *p*(ω, α) be the prior density of ω and α. The goal of Bayesian approach is to obtain statistical estimates from the joint posterior distribution *p*(κ, α | *Y*)∝*p*(κ, α)*p*(*Y* | κ, α). Given that, $Y^{*}=\left[y_{1}^{*},\ldots ,y_{n}^{*}\right]$ is the *p* × *n* matrix of latent measurements underlying *Y*, the values of *Y* can be augmented with Y^*^ in the posterior analysis. The Gibbs sampler is used to generate values for κ, α, and Y^*^. The joint posterior distribution *p*(κ, α, Y^*^ | *Y*) will be analyzed based on these values. Initial starting values [κ^(0)^, α^(0)^, Y^*(0)^] are used to simulate [κ^(1)^, α^(1)^, Y^*(1)^] in the next iteration and so on. Using the *j*th iteration with values κ^(*j*)^, α^(*j*)^, Y^*(*j*)^,

Step (a): Generate α^(*j*+1)^ from *p*(α | *Y*, κ^(*j*)^, Y^*(*j*)^);
Step (b): GenerateY^*(*j*+1)^ from *p*(Y^*^ | *Y*, κ^(*j*)^, α^(*j*+1)^);
Step (c): Generate κ^(*j*+1)^ from *p*(κ | *Y*, α^(*j*+1)^, Y^*(*j*+1)^)

As *j* approaches infinity, the statistical estimates of the joint density of [κ^(*j*)^, α^(*j*)^, Y^*(*j*)^] are said to approach those of the actual joint posterior density (κ, α, Y^*^ | *Y*). In order to eliminate the effect of the starting value, estimates from the first few iterations are discarded or allowed to “burn-in.”

### Simulation

Data was generated according to the ordinal CFA model specified in Equation (2). The study design was 2 (factor score distribution shapes: multivariate normal, multivariate mild skewed) × 3 (latent variable correlations: 0.2, 0.5, 0.8) × 6 (sample sizes: 42, 63, 84, 105, 210, 315) × 6 (estimation methods: Bayesian informative prior, Bayesian relatively less informative prior, RML, WLS, RDWLS, RULS) yielding six sets of estimates for each of the 36 conditions per dataset. These two priors for factor correlations [ξ ~ *Unif*(−1, 1), ξ ~ *Unif*(0, 1)] were chosen because the first prior represents the most pessimistic belief about the correlation. It is relatively less informative because it specifies that the correlation lies between −1 and +1, which are the mathematically possible boundaries. The second prior is a little more informative because the researcher (for substantive reasons) believes that factors are positively correlated.

The response data consisted of two factors with five items per factor. The items were measured on a four-point Likert scale. Factor pattern coefficients were generated from a uniform distribution ranging from 0.4 to 0.8. For multivariate normality, the factor scores were generated from unit normal distributions using Cholesky decomposition of the specified correlations. For multivariate non-normality, both traits were generated from a mildly skewed distribution (mean, 0; standard deviation, 1; skewness, 1.5; kurtosis, 1.5). Vale and Maurelli's (1983) algorithm was used to generate multivariate non-normal data. Five hundred replications of 36 fully crossed conditions were generated. Bayesian priors were specified as follows:

$$\begin{array}{l} a_{i}\sim N\left(0,1\right)I\left(0,\right);b_{i,c}\sim N\left(0,1\right)\;where\;b_{i,c-1}<b_{i,c};\\ {mu}_{j}\sim N\left(0,\;1\right)\\ \omega_{2p}\sim N_{2}\left(mu,\Sigma \right);\\ \Sigma =\left[\begin{array}{cc} 1 & \xi \\ \xi & 1 \end{array}\right]\\ \xi \sim \;Unif\left(-1,1\right)\text{ or }\xi \;\sim \text{ Unif}\left(0,1\right)depending\;on\;the\;prior \end{array}$$

In the specifications given above, *a*_*i*_ is the factor pattern coefficient and *b*_*i, c*_ is the threshold parameter for category *c* and item *i*, ω_2_ is the vector of two factor scores of the *p*-th person with mean vector *mu* (*j* = 2), covariance matrix Σ, and factor correlation ξ. The threshold parameters for a given item *i* were sorted in increasing order to ensure that the first threshold was smaller than the second threshold for a given item. The prior for factor loadings was a truncated normal distribution that was restricted to be positive. Convergence diagnostics such as multivariate potential scale reduction factor (MPSRF, Brooks and Gelman, 1998) and Heidelberger and Welch (1983) test indicated convergence with 2000 updates after 10,000 burn-ins for both prior specifications. Therefore, these parameters were used for Bayesian estimation. JAGS and LISREL were used for Bayesian (MCMC) and non-Bayesian estimation, respectively (see Appendix for codes). The present study only investigated factor correlations in order to focus on the estimates in greater detail.

Confidence intervals for latent variable correlations obtained from RML, WLS, RDWLS, and RULS were computed using Fisher's z transformation. Credibility intervals for Bayesian estimates were obtained from the posterior distribution and EAP scores (i.e., posterior means) were compared against non-Bayesian estimates. Root mean square error was computed as:

(7) $$\begin{array}{r} RMSE\left(\xi \right)\;=\;\sqrt{\frac{1}{R}\overset{R}{\underset{i=1}{\sum }}{\left({\xi .est}_{i}-\xi .true\right)}^{2}}, \end{array}$$

where, *R* is the total number of replications, ξ.*est*_*i*_is the parameter estimate for the *i*-th replication and ξ.*true* is the true value of the parameter ξ as specified by the study design. Bias was computed as:

(8) $$\begin{array}{r} Bias\left(\xi \right)=\frac{1}{R}\overset{R}{\underset{i=1}{\sum }}\left({\xi .est}_{i}-\xi .true\right). \end{array}$$

Negative interval bias was computed as the percentage of intervals that did not contain the true parameter value with both ends of the interval below the true parameter value. Similarly, positive interval bias was computed as the percentage of intervals that did not contain the true parameter value with both ends of the interval above the true parameter value. Width of the interval was computed as the difference between the upper end of the interval and the lower end of the interval.

The number of non-convergences and inadmissible solutions were counted for all estimates. Bayesian convergence was determined based on whether the 0.975th percentile value of the MPSRF was less than 1.2 (Brooks and Gelman, 1998). RMSE and bias for non-Bayesian estimates were computed after removing the non-converging and inadmissible solutions. That is, *R* in Equations (9) and (10) represents the number of solutions that were both converging and admissible. Finally, effect sizes from factorial ANOVAs (η^2^) were computed to only detect patterns in the results and not interpret statistical significance. The dependent variables were coverage, width, and positive and negative biases of interval estimates, and RMSE and bias of point estimates of the factor correlation. The independent variables were method (of estimation), sample size, correlation (between factor scores), and distribution (of factor scores). The ANOVA was a fully crossed 2 × 3 × 6 × 6 design with two factor score distributions (multivariate normal and multivariate mildly skewed), three correlations (ξ.*true* = 0.2, 0.5, 0.8), six sample sizes (*n* = 42, 63, 84, 105, 210, *and* 315), and six estimation methods (Bayesian-relatively less informative priors, Bayesian-informative priors, RML, RDWLS, RULS, and WLS). Following Cohen (1988), effect sizes were characterized as small, medium, and large when they were <1%, around 8%, and >14%, respectively.

In order to investigate the accuracy of standard errors, standard deviations of point estimates were compared with the average standard errors across all conditions (Carsey and Harden, 2014). Standard deviations of the point estimates represent the standard deviation of the sampling distribution of the point estimates. When standard deviations of point estimates are larger than the average standard errors, standard errors are underestimated. Similarly, when standard deviations of point estimates are smaller than the average standard errors, standard errors are overestimated. Ideally, both these statistics should be close in value.

## Results

All non-Bayesian methods produced 0.2–61.4% inadmissible solutions and/or ran into non-convergence issues for *n* ≤ 105 (Table 1). WLS estimates could not be computed for any of the 500 replications when *n* = 42 because of non-positive definite asymptotic covariance matrices. WLS also ran into estimation issues more often than other methods. Following WLS, RML had the highest percentage of inadmissible and non-converging solutions, and especially so for non-normal factor scores. In general, the percentage of non-convergence and inadmissibility decreased with an increase in sample size. Non-convergence and inadmissibility was higher for non-normal factor scores. RDWLS and RULS produced fewest non-converging and inadmissible solutions. MPSRF values for all Bayesian estimates were less than 1.2 for all datasets, indicating support for convergence.

**Table 1:** **Non-converging and inadmissible solutions for RML, RDWLS, RULS, and WLS in percentages**.

***n***	**Dist**	**WLS**		**RULS**		**RDWLS**		**RML**	
		**Inadm**	**Noncon**	**Inadm**	**Noncon**	**Inadm**	**Noncon**	**Inadm**	**Noncon**
42	Normal	^**^	^**^	9.8	1.8	12.2	2.2	29.4	21.6
	Skewed	^**^	^**^	18	3.4	21.4	3.8	38.6	21
63	Normal	21.6	4.2	8.2	0.4	7.6	0	13	4
	Skewed	23.8	4.8	8.6	0.4	9.4	0.2	14.2	4.8
84	Normal	6.6	1.6	1.2	0	1	0	2	0.6
	Skewed	11.6	1.8	6.4	0	5.4	0.2	7.2	0.8
105	Normal	3.8	0.4	1.2	0	1.4	0.2	1.4	0.4
	Skewed	6.6	0.2	3.2	0	3.6	0	3.2	0.2
210	Normal	0.2	0	0	0	0	0	0	0
	Skewed	0.6	0	0	0	0	0	0	0
315	Normal	0	0	0	0	0	0	0	0
	Skewed	0	0	0	0	0	0	0	0

*inadm = percentage of inadmissible solutions; noncon = percentage of non-converging solutions*;

** *non-positive definite asymptotic covariance matrix, therefore no solutions*.

RMSE, bias, coverage, width, and positive and negative biases are compared across only converging and admissible estimates. Therefore, the ANOVA results in Table 2 need to be interpreted bearing in mind that the performance of non-Bayesian methods (especially WLS) seem more efficient than they actually were. Tables 3–6 show the coverage rates, width, RMSE, and bias of the estimation methods by sample size and factor score correlations for normally and non-normally distributed factor scores, respectively.

**Table 2:** **Effect sizes (η^2^) from Factorial ANOVAs in percentages for converging and admissible solutions**.

**Effects**	**Coverage**	**Width**	**Negative interval bias**	**Positive interval bias**	**RMSE**	**Bias**
Method	60.24	16.40	63.69	61.61	13.24	43.58
*n*		34.82			61.63	
Distribution						
Factor correlation	11.23	21.34		9.66	8.67	
Method × *n*					9.55	12.47
Method × Factor correlation			10.87			13.10

**Table 3:** **Coverage rates for converging and admissible estimates**.

***n***	**ξ**	**Distributions**											
		**Multivariate Normal**						**Multivariate Mildly Skewed**					
		**Bayesian**		**Non-Bayesian**				**Bayesian**		**Non-Bayesian**			
		**inf**	**lessinf**	**WLS**	**RULS**	**RDWLS**	**RML**	**inf**	**lessinf**	**WLS**	**RULS**	**RDWLS**	**RML**
42	0.2	1.000	0.956	^**^	0.721	0.703	0.756	0.970	0.938	^**^	0.732	0.718	0.770
	0.5	0.970	0.966	^**^	0.742	0.721	0.710	0.970	0.952	^**^	0.693	0.646	0.680
	0.8	0.920	0.930	^**^	0.617	0.604	0.549	0.970	0.952	^**^	0.575	0.578	0.560
63	0.2	0.950	0.948	0.467	0.779	0.768	0.781	0.960	0.962	0.467	0.745	0.743	0.772
	0.5	0.970	0.962	0.324	0.747	0.736	0.722	0.930	0.944	0.388	0.697	0.687	0.710
	0.8	0.920	0.932	0.337	0.585	0.573	0.538	0.940	0.958	0.334	0.548	0.552	0.521
84	0.2	0.970	0.938	0.478	0.756	0.760	0.783	0.970	0.960	0.523	0.764	0.762	0.793
	0.5	0.930	0.950	0.473	0.784	0.776	0.794	0.950	0.938	0.410	0.715	0.705	0.693
	0.8	0.970	0.942	0.380	0.573	0.568	0.525	0.900	0.960	0.336	0.565	0.533	0.557
105	0.2	0.970	0.944	0.579	0.788	0.792	0.798	0.950	0.928	0.541	0.760	0.760	0.762
	0.5	0.920	0.940	0.486	0.722	0.720	0.727	0.960	0.966	0.492	0.740	0.720	0.750
	0.8	0.940	0.946	0.446	0.593	0.589	0.590	0.950	0.930	0.402	0.550	0.546	0.527
210	0.2	0.950	0.942	0.714	0.820	0.818	0.824	0.990	0.948	0.690	0.774	0.772	0.778
	0.5	0.930	0.944	0.662	0.762	0.766	0.744	0.940	0.948	0.616	0.754	0.746	0.752
	0.8	0.880	0.914	0.467	0.582	0.578	0.558	0.990	0.922	0.404	0.530	0.522	0.514
315	0.2	0.980	0.956	0.780	0.824	0.822	0.820	0.960	0.952	0.736	0.822	0.820	0.828
	0.5	0.960	0.952	0.700	0.748	0.754	0.754	0.940	0.958	0.636	0.744	0.742	0.748
	0.8	0.950	0.926	0.518	0.606	0.618	0.620	0.850	0.906	0.402	0.466	0.472	0.464

** *non-positive definite asymptotic covariance matrix*;

*inf = informative priors; lessinf = relatively less informative priors; ξ = Factor Correlation*.

**Table 4:** **Widths by estimation method, sample size, factor correlations, and factor score distributions for converging and admissible estimates**.

***n***	**ξ**	**Distributions**											
		**Multivariate Normal**						**Multivariate Mildly Skewed**					
		**Bayesian**		**Non-Bayesian**				**Bayesian**		**Non-Bayesian**			
		**inf**	**lessinf**	**WLS**	**RULS**	**RDWLS**	**RML**	**inf**	**lessinf**	**WLS**	**RULS**	**RDWLS**	**RML**
42	0.2	0.536	0.788	^**^	0.544	0.541	0.557	0.538	0.796	^**^	0.537	0.532	0.549
	0.5	0.633	0.718	^**^	0.426	0.420	0.455	0.637	0.796	^**^	0.402	0.396	0.432
	0.8	0.522	0.524	^**^	0.218	0.213	0.251	0.490	0.796	^**^	0.205	0.206	0.242
63	0.2	0.481	0.657	0.396	0.458	0.457	0.465	0.484	0.668	0.395	0.452	0.451	0.457
	0.5	0.548	0.579	0.257	0.354	0.352	0.371	0.555	0.668	0.266	0.347	0.343	0.364
	0.8	0.405	0.417	0.120	0.179	0.176	0.200	0.410	0.668	0.114	0.157	0.155	0.177
84	0.2	0.448	0.570	0.366	0.397	0.396	0.401	0.448	0.579	0.368	0.397	0.397	0.401
	0.5	0.483	0.506	0.258	0.315	0.312	0.324	0.495	0.579	0.248	0.304	0.302	0.316
	0.8	0.375	0.369	0.112	0.152	0.148	0.163	0.352	0.579	0.103	0.137	0.132	0.149
105	0.2	0.408	0.517	0.343	0.360	0.359	0.362	0.425	0.516	0.340	0.357	0.356	0.359
	0.5	0.437	0.453	0.241	0.279	0.277	0.286	0.463	0.516	0.239	0.275	0.273	0.283
	0.8	0.330	0.330	0.107	0.138	0.136	0.145	0.316	0.516	0.099	0.125	0.124	0.132
210	0.2	0.324	0.368	0.254	0.257	0.256	0.257	0.337	0.378	0.252	0.255	0.255	0.256
	0.5	0.320	0.325	0.190	0.201	0.201	0.204	0.329	0.378	0.188	0.199	0.198	0.201
	0.8	0.229	0.228	0.085	0.097	0.096	0.099	0.226	0.378	0.077	0.085	0.085	0.087
315	0.2	0.282	0.303	0.209	0.211	0.211	0.211	0.286	0.306	0.209	0.210	0.210	0.211
	0.5	0.262	0.263	0.159	0.165	0.165	0.166	0.267	0.306	0.156	0.162	0.162	0.163
	0.8	0.196	0.190	0.073	0.079	0.079	0.080	0.183	0.306	0.065	0.070	0.070	0.071

** *non-positive definite asymptotic covariance matrix*;

*inf = informative priors; lessinf = relatively less informative priors; ξ = Factor Correlation*.

**Table 5:** **RMSE by estimation method, sample size, factor correlations, and factor score distributions for converging and admissible estimates**.

***n***	**ξ**	**Distributions**											
		**Multivariate Normal**						**Multivariate Mildly Skewed**					
		**Bayesian**		**Non-Bayesian**				**Bayesian**		**Non-Bayesian**			
		**inf**	**lessinf**	**WLS**	**RULS**	**RDWLS**	**RML**	**inf**	**lessinf**	**WLS**	**RULS**	**RDWLS**	**RML**
42	0.2	0.116	0.206	^**^	0.267	0.277	0.243	0.135	0.213	^**^	0.277	0.284	0.261
	0.5	0.139	0.188	^**^	0.226	0.269	0.246	0.151	0.179	^**^	0.236	0.290	0.259
	0.8	0.160	0.146	^**^	0.208	0.266	0.221	0.117	0.139	^**^	0.215	0.215	0.201
63	0.2	0.131	0.169	0.380	0.206	0.213	0.196	0.111	0.170	0.369	0.208	0.209	0.200
	0.5	0.154	0.150	0.294	0.168	0.186	0.180	0.142	0.157	0.281	0.192	0.207	0.206
	0.8	0.121	0.122	0.152	0.112	0.176	0.192	0.103	0.107	0.191	0.104	0.129	0.147
84	0.2	0.110	0.149	0.282	0.173	0.175	0.168	0.108	0.148	0.277	0.173	0.174	0.167
	0.5	0.135	0.130	0.210	0.135	0.135	0.141	0.127	0.138	0.234	0.142	0.144	0.154
	0.8	0.100	0.104	0.111	0.093	0.093	0.107	0.097	0.092	0.178	0.118	0.145	0.152
105	0.2	0.099	0.138	0.218	0.151	0.151	0.148	0.113	0.146	0.234	0.160	0.161	0.158
	0.5	0.125	0.125	0.187	0.136	0.133	0.131	0.110	0.113	0.194	0.121	0.131	0.131
	0.8	0.094	0.089	0.100	0.082	0.082	0.090	0.074	0.087	0.124	0.083	0.110	0.117
210	0.2	0.083	0.097	0.121	0.103	0.103	0.101	0.077	0.097	0.126	0.105	0.105	0.104
	0.5	0.092	0.087	0.107	0.087	0.087	0.089	0.081	0.083	0.106	0.085	0.085	0.087
	0.8	0.070	0.066	0.066	0.064	0.062	0.066	0.063	0.062	0.076	0.069	0.067	0.069
315	0.2	0.066	0.077	0.088	0.080	0.081	0.080	0.074	0.076	0.088	0.078	0.079	0.077
	0.5	0.065	0.069	0.078	0.070	0.069	0.070	0.073	0.067	0.084	0.070	0.071	0.070
	0.8	0.050	0.053	0.052	0.049	0.049	0.050	0.063	0.055	0.067	0.060	0.060	0.060

** *non-positive definite asymptotic covariance matrix*;

*inf = informative priors; lessinf = relatively less informative priors; ξ = Factor correlation*.

**Table 6:** **Bias by estimation method, sample size, factor correlations, and factor score distributions for converging and admissible estimates**.

***n***	**ξ**	**Distributions**											
		**Multivariate Normal**						**Multivariate Mildly Skewed**					
		**Bayesian**		**Non-Bayesian**				**Bayesian**		**Non-Bayesian**			
		**inf**	**lessinf**	**WLS**	**RULS**	**RDWLS**	**RML**	**inf**	**lessinf**	**WLS**	**RULS**	**RDWLS**	**RML**
42	0.2	0.060	−0.039	^**^	0.003	0.006	−0.023	0.057	−0.026	^**^	0.019	0.030	−0.010
	0.5	−0.026	−0.061	^**^	0.016	0.004	−0.043	−0.025	−0.025	^**^	0.050	0.032	−0.013
	0.8	−0.089	−0.075	^**^	−0.014	−0.027	−0.052	−0.034	−0.061	^**^	−0.003	−0.005	−0.037
63	0.2	0.050	−0.031	0.062	−0.008	−0.008	−0.033	0.045	−0.007	0.084	0.023	0.028	0.005
	0.5	−0.042	−0.033	0.157	0.019	0.017	−0.02	−0.028	−0.026	0.145	0.026	0.026	−0.015
	0.8	−0.040	−0.048	0.065	−0.001	−0.008	−0.043	−0.028	−0.027	0.065	0.028	0.026	−0.004
84	0.2	0.040	−0.004	0.081	0.017	0.020	0.001	0.038	−0.009	0.072	0.015	0.017	−0.003
	0.5	−0.043	−0.036	0.115	0.006	0.013	−0.017	−0.016	−0.017	0.126	0.030	0.035	−0.002
	0.8	−0.042	−0.041	0.059	0.004	0.009	−0.014	−0.021	−0.020	0.060	0.023	0.025	−0.001
105	0.2	0.028	−0.019	0.049	0.003	0.005	−0.012	0.052	−0.004	0.054	0.017	0.018	0.002
	0.5	−0.024	−0.023	0.094	0.011	0.016	−0.006	−0.015	−0.014	0.098	0.025	0.027	0.002
	0.8	−0.034	−0.033	0.049	0.000	0.003	−0.012	−0.012	−0.014	0.057	0.021	0.020	0.007
210	0.2	0.001	−0.004	0.024	0.007	0.007	0.000	0.020	0.006	0.038	0.017	0.018	0.010
	0.5	−0.021	−0.016	0.040	0.003	0.005	−0.008	−0.005	−0.008	0.050	0.013	0.015	0.003
	0.8	−0.017	−0.015	0.028	0.002	0.003	−0.004	0.004	0.006	0.046	0.028	0.029	0.023
315	0.2	−0.003	−0.007	0.013	0.000	0.000	−0.005	0.016	−0.002	0.016	0.005	0.006	0.001
	0.5	−0.011	−0.011	0.025	0.001	0.002	−0.005	0.009	0.001	0.041	0.015	0.016	0.009
	0.8	−0.017	−0.008	0.019	0.002	0.003	−0.002	0.009	0.011	0.041	0.027	0.028	0.023

** *non-positive definite asymptotic covariance matrix*;

*inf = informative priors; lessinf = relatively less informative priors; ξ = Factor correlation*.

### Coverage

**Figure 1:**
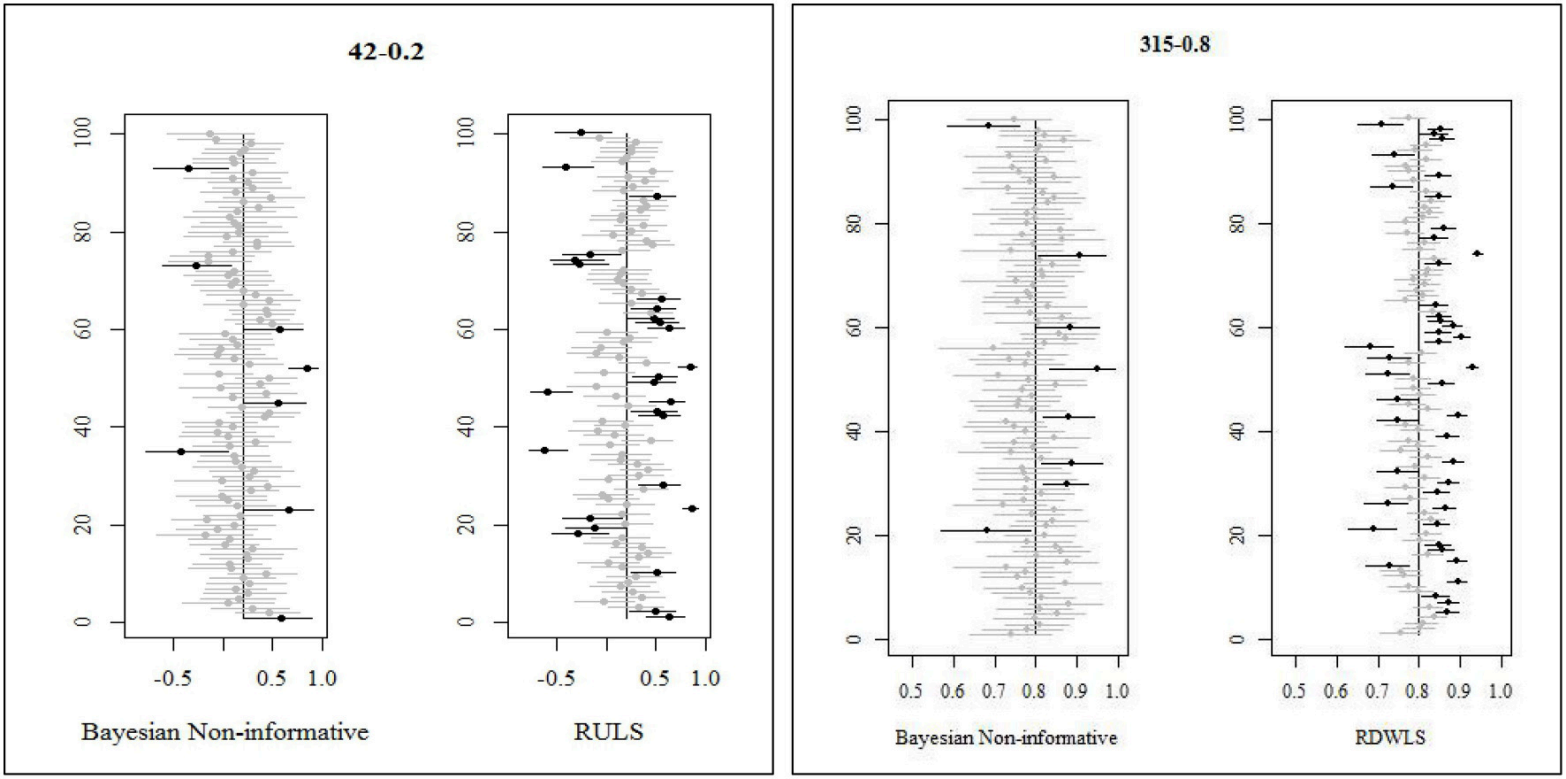
**Coverage rates of Bayesian, RULS, and RDWLS for 100 randomly selected replications**.

Method of estimation and ξ.*true* values explained 60% and 11% of the variation in coverage, respectively (Table 2). Bayesian informative priors had the best coverage followed closely by Bayesian relatively less informative priors. RDWLS, RML, and RULS coverages were comparable to each other, but these were 12–45% less than Bayesian coverage (Table 3). WLS had the least coverage with 17–63% less than Bayesian coverage. For Bayesian relatively less informative priors, 91.6–96.6% of the credibility intervals contained the true value. For Bayesian informative priors 88.8–100% of the credibility intervals contained the true value. For ξ.*true* = 0.8, Bayesian informative prior credibility intervals were 90 and –85% for normal and mild skewed factor correlations and *n* = 210 *and* 315, respectively. Bayesian informative priors had slightly less coverage than Bayesian relatively less informative priors (≤ 6%) for some cases of larger samples when ξ.*true* = 0.5, 0.8. Considering only the converging and admissible estimates, WLS CI coverage was 32–78% (for *n* ≥ 63); RULS CI coverage was 57–82%; RML CI coverage was 52–82%; RDWLS CI coverage was 56–82%. WLS CI coverage was 33–74% (for *n* ≥ 63); RULS CI coverage was 47–82%; RML CI coverage was 46–83%; RDWLS CI coverage was 47–82% for non-normal factor scores. For high factor correlations (ξ.*true* = 0.8) both Bayesian and non-Bayesian intervals had higher undercoverage. Especially for mildly skewed distributions and ξ.*true* = 0.8, non-Bayesian methods had severe undercoverage (40–58%). There were no cases of overcoverage for the non-Bayesian approaches, whereas Bayesian informative prior credibility intervals had slight overcoverage. Figure 1 shows interval estimates of a randomly selected sample of 100 replications for Bayesian relatively less informative, RULS, and RDWLS. The point estimates are indicated by dots, and dots in gray correspond to the gray line intervals that captured the true value. Dots and lines in black failed to capture the true value. In Figure 1 Bayesian estimates have fewer black lines than RWLS.

### Width

**Figure 2:**
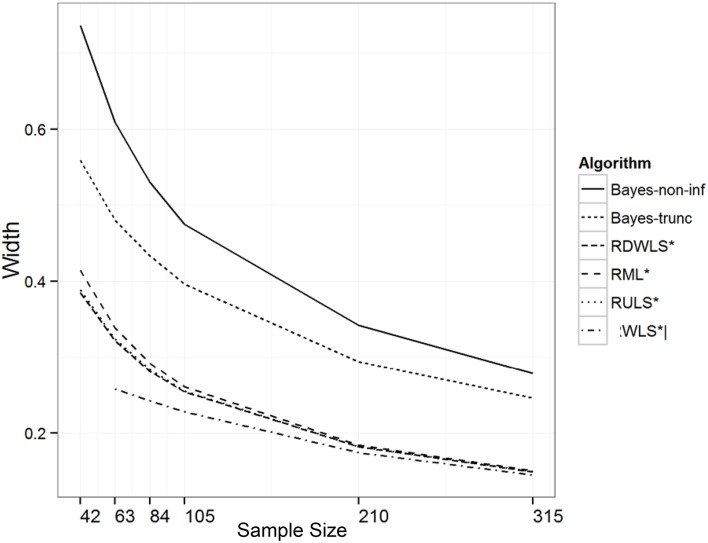
**Width of intervals by algorithm and sample size (trunc = informative prior)**. ^*^only converging and admissible estimates used.

Sample size, ξ.*true* values, and method of estimation explained 35%, 21%, and 16% of the variation in the width of the intervals, respectively. As expected, the width of the intervals decreased with an increase in sample size (Table 4). The width of the intervals also decreased with an increase in ξ.*true* values. This is a possible reason for undercoverage at higher correlations. Bayesian relatively less informative priors had the widest intervals followed by Bayesian informative priors. RML, RULS, RDWLS, and WLS intervals were shorter than Bayesian intervals (Figure 2). Wider credibility intervals are one reason for higher coverage of Bayesian methods. RML, RULS, and RDWLS widths were comparable.

### Positive interval bias

**Figure 3:**
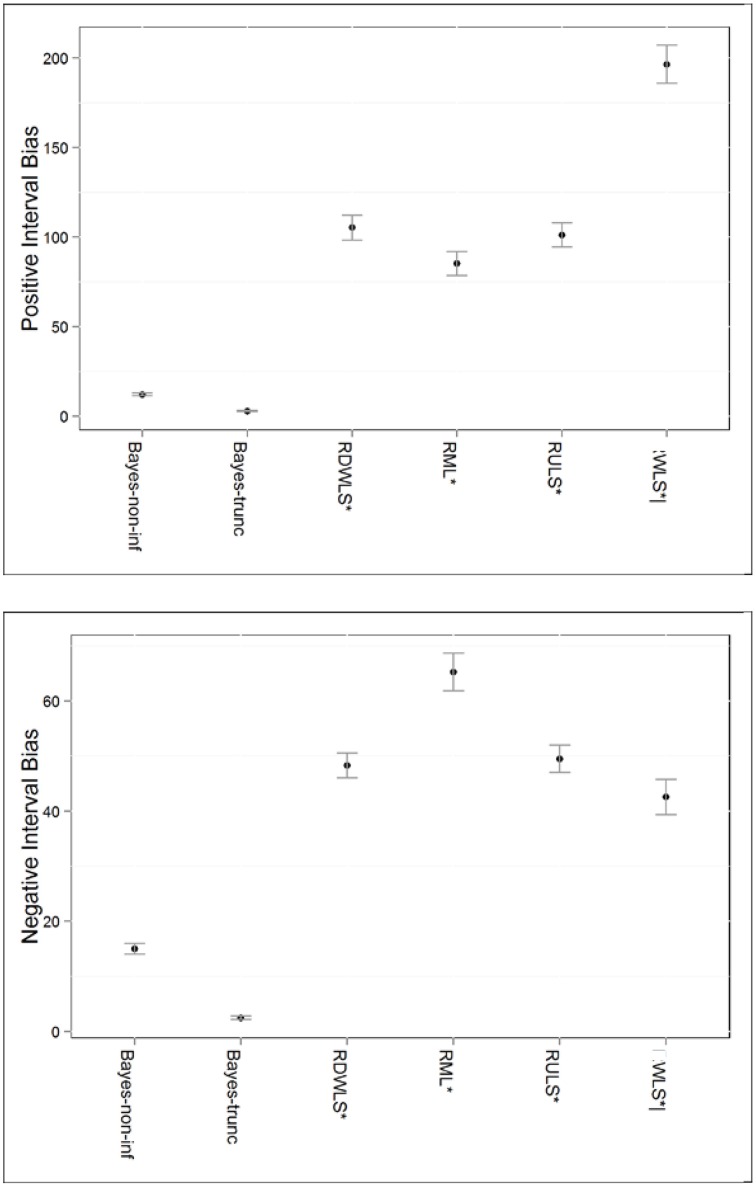
**Positive and negative interval bias by estimation method**. ^*^Only converging estimates were used to compute interval biases. RMLS, RDWLS, RULS, and WLS estimates not directly comparable to Bayesian estimates; |Only *n* ≥ 63 estimates are represented for WLS.

Method of estimation and ξ.*true* explained 61.6% and 9.6% of the variation in positive bias of interval estimates, respectively. Bayesian intervals had the least positive bias as shown in Figure 3. This is not too surprising given that most Bayesian intervals contained the true value of the parameter. This was followed by RML, RULS, and RDWLS. Up to 57% of the converging WLS interval estimates for *n* ≥ 63 were positively biased.

### Negative interval bias

Method of estimation, and the interaction effect of method and ξ.*true* explained 63.6% and 10.8% of the variation in negative bias of interval estimates, respectively. Again, both sets of Bayesian interval estimates had the least negative bias. The patterns for negative and positive interval bias were similar with the exception of: (a) RML intervals having more negative bias but less positive bias than RDWLS and RULS (Figure 3), and (b) more intervals being positively biased than negatively biased. More negative bias occurred in RDWLS, RML, and RULS intervals for higher ξ.*true* values.

### Standard errors

The means of the standard errors (μ.*se*) were compared with the empirical standard errors, that is, the standard deviations of the point estimates (σ.μ). Table 7 shows that all non-Bayesian estimates had larger empirical standard errors than average standard errors, especially for smaller samples. RDWLS, RML, and RULS empirical standard errors were up to three times larger than the average standard errors. This shows that the standard error estimates of non-Bayesian methods are severely underestimated even after using ACMs. Bayesian standard error estimates (posterior standard deviations) were closer to their empirical standard errors (standard deviation of posterior means). Bayesian estimates for both priors were combined in Table 7 because they were identical up to the second decimal place.

**Table 7:** **Means of standard errors and standard deviations of means by estimation method, sample size, and factor correlations**.

**ξ**	***n***	**Bayesian**		**RDWLS**		**RML**		**RULS**		**WLS**	
		**μ.s*e***	**σ.μ**	**μ.s*e***	**σ.μ**	**μ.s*e***	**σ.μ**	**μ.s*e***	**σ.μ**	**μ.s*e***	**σ.μ**
0.2	42	0.193	0.204	0.13	0.280	0.138	0.252	0.135	0.272	0.139	0.257
	63	0.162	0.165	0.11	0.211	0.115	0.198	0.114	0.207	0.099	0.368
	84	0.141	0.145	0.10	0.174	0.100	0.167	0.099	0.173	0.092	0.269
	105	0.127	0.141	0.09	0.156	0.090	0.153	0.090	0.155	0.085	0.221
	210	0.093	0.096	0.06	0.104	0.064	0.102	0.064	0.104	0.063	0.120
	315	0.076	0.076	0.05	0.080	0.053	0.078	0.053	0.079	0.052	0.087
0.5	42	0.173	0.170	0.10	0.279	0.111	0.251	0.103	0.229	0.114	0.254
	63	0.143	0.146	0.09	0.196	0.092	0.192	0.088	0.179	0.065	0.245
	84	0.125	0.126	0.08	0.137	0.080	0.147	0.077	0.138	0.063	0.187
	105	0.113	0.121	0.07	0.130	0.071	0.131	0.069	0.127	0.060	0.165
	210	0.081	0.086	0.05	0.085	0.051	0.088	0.050	0.086	0.047	0.097
	315	0.066	0.068	0.04	0.069	0.041	0.070	0.041	0.070	0.039	0.074
0.8	42	0.130	0.126	0.05	0.242	0.062	0.207	0.053	0.211	0.070	0.199
	63	0.104	0.111	0.04	0.155	0.047	0.169	0.042	0.107	0.029	0.160
	84	0.092	0.095	0.03	0.121	0.039	0.131	0.036	0.105	0.027	0.135
	105	0.082	0.083	0.03	0.096	0.035	0.104	0.033	0.082	0.026	0.099
	210	0.057	0.065	0.02	0.063	0.023	0.067	0.023	0.064	0.020	0.061
	315	0.047	0.053	0.02	0.052	0.019	0.054	0.019	0.053	0.017	0.052

*μ.se = Mean of Standard Errors; σ.μ = Standard Deviation of Means; ξ = Factor correlation*.

### Point estimate RMSE

Sample size, method of estimation, ξ.*true*, and the interaction between method and sample size explained 62%, 13%, 9%, and 9.5% of the variation in RMSE of point estimates, respectively. The RMSE decreased with an increase in the sample size, as expected. Bayesian informative prior RMSEs were the lowest and were 52–56% lower than RML, RULS, and RDWLS. RMSEs for Bayesian informative priors were 30–45% lower than RML, RULS, and RDWLS. Between the two Bayesian priors, the RMSEs for informative priors were up to 78% lower than those of relatively less informative priors. However, most of these RMSEs were in the range of 0.05–0.28 and the differences were to the second decimal. WLS had the highest marginal RMSE, even when estimates for only *n* ≥ 63 were included. The difference between the RMSEs among all methods decreased with an increase in sample size and the RMSEs were comparable for all methods when *n* ≥ 210. Higher values of ξ.*true* were associated with larger RMSEs.

### Point estimate bias

Method of estimation, and the interaction effects between method and sample size and method and ξ.*true* explained 43.6%, 12.5%, and 13% of the variation in the bias of point estimates, respectively. In general, the bias decreased with an increase in the sample size. The change was more prominent for WLS, followed by Bayesian relatively less informative priors, and RML. WLS estimates were the most and positively biased. On average, Bayesian and RML were negatively biased while the rest were slightly positively biased.

## Limitations

The present study considered only a two-factor model which is the simplest CFA model. However, the priors of the CFA models with more factors will need careful parametrizing to avoid running into non-positive definite covariance matrices. It is unclear how the prior specification for factor covariance matrices with higher order (e.g., 3 × 3 or 4 × 4) will impact the credibility interval estimates.

Due to the computationally intensive nature of the simulation and the time taken for Bayesian estimation, only the intervals of factor correlations were compared but not the other parameters. Therefore, the results of the present study cannot be generalized to the interval estimates of all CFA parameters. Only the priors for factor correlations were varied in the present study in order to control confounding effects on the joint posterior probabilities. Obviously, varying all prior specifications across conditions will impact the final estimates. These are avenues for further research.

## Conclusion

Although RMSEs and bias of point estimates showed fewer advantages to Bayesian estimation, analysis of interval estimates showed clearer advantages to Bayesian estimation. Eighty-five to hundred percent of the Bayesian credibility intervals contained the true parameter value but coverage of non-Bayesian estimates was lower for even the converging and admissible solutions. WLS, RULS, RDWLS, and RML interval estimates contained only 32–78% (for *n* ≥ 63), 46–82%, 47–82%, and 46–83% of the true parameter values, even with samples as large as 105 (5df). The coverage of non-Bayesian methods was severely affected by the non-normality of the factor scores for large samples with large ξ.*true* values, but to a very small extent for other data conditions. WLS estimates could not be obtained for *n* = 42 due to non-positive definite asymptotic covariance matrices. Moreover, WLS performed the worst for all conditions and with respect to all diagnostics of interval and point estimates considered in the present study. This confirms Wothke's (1993) and Yang-Wallentin et al.'s (2010) suggestions about the unsuitability of WLS for small samples.

Similar to the findings of previous studies (e.g., DiStefano, 2002; Yang-Wallentin et al., 2010; Bandalos, 2014), non-Bayesian methods underestimated standard errors, especially for small samples. These methods need to apply corrections for standard error estimation for small samples. Although, smaller standard errors may be more desirable, underestimated standard errors result in inflated Type-I errors. This is evident in the severe undercoverage of non-Bayesian methods.

Some Bayesian intervals had overcoverage, but in general, most 95% credibility intervals had about 95% coverage. Bayesian intervals for smaller samples compensated for the sample size by including more uncertainty than non-Bayesian methods. Non-Bayesian intervals had more positive bias than negative bias, that is, the confidence interval lower limits were higher than the true parameter value.

Higher ξ.*true* values were recovered less accurately than lower values (point RMSE and coverage) because large true correlations allow more discrepancies between true and estimated correlations (Kline, 2010). All methods showed least recovery of factor correlations for high ξ.*true* values. Bayesian and non-Bayesian methods produced similar estimates of factor correlations, with very slight advantage to Bayesian methods for small samples as shown by the RMSEs. Weighted least squares estimates had the highest RMSE and bias, making it the least suited technique for ordinal CFA with small samples.

Using an informative prior increased the accuracy of the point estimates (RMSE and bias) and reduced the width of the credibility interval, while still retaining coverage. This shows clear advantage to using priors in a systematic manner to improve Bayesian estimation. For instance, information about a parameter may be collected through meta-analysis or systematic literature review, so appropriate prior distributions could be specified. It should be noted here that the priors used for the rest of the parameters were mildly informative. Having priors that are informative can help speed up convergence. Given that the use of appropriate priors is a major advantage of Bayesian estimation, the present study further goes to show how even mildly informative priors can be used effectively to obtain estimates for small samples.

All non-Bayesian methods had several non-converging and inadmissible solutions for small samples *n* ≤ 105. These results confirm the findings of Bandalos (2014), Yang-Wallentin et al. (2010) and Yuan et al. (2011). The results of the present study need to be interpreted with caution, because the 500 converging Bayesian estimates were compared with 193–494 converging and admissible non-Bayesian estimates. Therefore, the performance of all non-Bayesian estimates is highly exaggerated except for *n* = 315.

The news may not be all positive for Bayesian because some of the intervals were up to 1.8 times wider than the non-Bayesian intervals for smaller samples. Extremely wide intervals need to be interpreted with caution because more information may be contributed by the prior than the data. This is due to the basic Bayesian proportionality formula where the posterior is proportional to the product of the likelihood (i.e., information contained in the data) and the prior. When less information is contained in the data (e.g., small samples), the prior has a stronger influence on the posterior. Therefore, credibility intervals need to be examined carefully, because in extreme cases they may provide no information beyond that provided by the prior.

There is no magical cure for lack of information. However, when a researcher is faced with small sample ordinal data Bayesian estimation provides reasonable point and interval estimates. Even when converging and admissible non-Bayesian point estimates have comparable RMSEs and biases, they severely underestimate standard errors. This affects the coverage of the confidence intervals and may lead to a higher probability of Type-I error. On the other hand, Bayesian priors must be chosen carefully. Future research may examine how prior information can be included in a systematic manner based on previous empirical research and posterior predictive model checks could be used for model misspecification problems.

In sum, the present study's results reveal the following three take-home messages: (a) even though Bayesian intervals contain more uncertainty because of the impact of priors in small samples they more accurately deliver their promise of probability allowing the researcher to make informed decisions, (b) comparison of point estimates and standard errors alone in simulation studies can be misleading, and therefore, (c) simulation studies should include comparisons of various interval diagnostics in order to understand the complete picture behind estimates.

### Conflict of interest statement

The author declares that the research was conducted in the absence of any commercial or financial relationships that could be construed as a potential conflict of interest.
