# Supplementary material for: Comparing interval estimates for small sample ordinal CFA models
Source: Front Psychol. 2015 Oct 30;6:1599. doi: 10.3389/fpsyg.2015.01599 (PMC4626630; doi:10.3389/fpsyg.2015.01599)
Supplement: Supplementary file 1 [file DataSheet1.DOCX]

**Appendix A**

**JAGS Code**

model { **#model for ordinal CFA begins here**

for (i in 1:N) {

for (j in 1:L) {

for (k in 1:(K-1)){

logit(p.star[i, j, k]) <- a[j]*(theta[i, 1] - b[j, k])

logit(p.star[i, j+L, k]) <- a[j+L]*(theta[i, 2] - b[j+L, k])

}

p[i, j, 1] <- 1 - p.star[i, j, 1]

p[i, j+L, 1] <- 1 - p.star[i, j+L, 1]

for (k in 2:(K - 1)) {

p[i, j, k] <- p.star[i,j, (k - 1)] - p.star[i, j, k]

p[i, j+L, k] <- p.star[i,j+L, (k - 1)] - p.star[i, j+L, k]

}

p[i, j, K] <- p.star[i, j, (K-1)]

p[i, j+L, K] <- p.star[i, j+L, (K-1)]

Y[i, j] ~ dcat(p[i, j, 1:K])

Y[i, (j + L)] ~ dcat(p[i, j+L, 1:K])

}

}

for (i in 1:N) { **#prior specifications begin here**

theta[i,1:2] ~ dmnorm(mu[1:2], tau[1:2,1:2])

}

mu[1] ~ dnorm(0,1)

mu[2] ~ dnorm(0,1)

Sigma2[1,1] <- 1

Sigma2[1,2] <- rho

Sigma2[2,1] <- rho

Sigma2[2,2] <- 1

tau[1:2,1:2] <- inverse(Sigma2)

rho ~ dunif(0,1)

**#Note: the above line needs to be changed for non-informative prior as dunif(-1,1)**

for (j in 1:2*L) {

a[j] ~ dnorm(1, 1)I(0, ) **#factor pattern coefficients**

for (k in 1:(K - 1)) {

b.star[j, k] ~ dnorm(0, 1) **#thresholds**

}

b[j, 1:(K - 1)] <- sort(b.star[j, 1:(K - 1)]) **#sort the thresholds**
} }

**LISREL CODE**

!Fitting a confirmatory factor analysis model in 2-factors

DA NI=10 NO=42 RP=500 MA=PM

LA

v1 v2 v3 v4 v5 v6 v7 v8 v9 v10

PM ='mildlis3_1.PM'

AC ='mildlis3_1.AM'

MO NX=10 NK=2

LK

fac1 fac2

FR LX(1,1) LX(2,1) LX(3,1) LX(4,1) LX(5,1) LX(6,2) LX(7,2) LX(8,2) LX(9,2) LX(10,2)

OU ME=UL ND=4 PV=parU3_1.pv

!Note: Change ME to WLS for weighted least squares, ML for robust maximum likelihood, and DWLS for diagonally weighted least squares
